# Hydrogel-Based Ocular Drug Delivery Systems: A Bibliometric and Visualization Analysis of Research Trends and Hotspots (1991–2025)

**DOI:** 10.3390/pharmaceutics18080981

**Published:** 2026-08-09

**Authors:** Linyang Li, Chen Huang, Xiaotong Yu, Ziyuan Liu

**Affiliations:** 1 Department of Ophthalmology, Peking University Third Hospital, Beijing 100191, China; 2210301238@stu.pku.edu.cn; 2 Peking University People’s Hospital, Beijing 100044, China; 3 Center of Basic Medical Research, Institute of Medical Innovation and Research, Peking University Third Hospital, Beijing 100191, China; yuxiaotong1617@163.com

**Keywords:** hydrogel, ocular drug delivery, bibliometric analysis, visual analysis, knowledge mapping, research trends

## Abstract

**Background**: Ocular drug delivery is hindered by the eye’s complex anatomy and multiple physiological barriers. Because of their biocompatibility, biodegradability, and capacity to prolong drug retention, hydrogels have emerged as promising platforms for ocular drug delivery. This study provides a bibliometric and visual analysis of research on hydrogel-based ocular drug delivery from 1991 to 25 August 2025. **Methods**: Publications were retrieved from the Web of Science Core Collection and screened for relevance. CiteSpace, VOSviewer, and the Bibliometrix R package were used to analyze annual publication trends, contributions and collaborations among countries, institutions, and authors, core journals, and evolving research themes. **Results**: A total of 1354 publications (991 articles and 363 reviews) were included. Annual output increased markedly after 2007–2008, reflecting rapid growth in this field. China and the USA were the leading contributors, and the University of Florida was the most productive institution. Keyword co-occurrence, clustering, and burst analyses identified in situ hydrogels, tissue engineering applications, and anti-VEGF delivery as major research hotspots. **Conclusions**: This study maps the growth and diversification of hydrogel-based ocular drug delivery research, revealing a shift toward disease- and segment-specific applications, with controlled-release, stimuli-responsive systems, injectable hydrogels, and anti-VEGF as emerging research priorities.

## 1. Introduction

The eye is a complex and delicate organ characterized by intricate anatomical structures and sophisticated physiological barriers. Ocular diseases are highly prevalent and pose a significant threat to quality of life worldwide [1,2,3]. While numerous potent therapeutic agents have been developed, their clinical efficacy is often compromised by suboptimal delivery to target ocular tissues. Topical formulations, especially eye drops, are still the most widely used ophthalmic dosage form. However, their effectiveness is constrained by rapid precorneal clearance, low corneal permeability, and limited bioavailability [4]. These limitations have driven the development of advanced ocular drug delivery systems with improved retention, penetration, and controlled release properties.

Hydrogels are three-dimensional, hydrophilic polymeric networks capable of absorbing and retaining substantial amounts of water while maintaining structural integrity [5,6]. Because of their high water content, soft-tissue-like properties, and adaptable physicochemical characteristics, hydrogels have emerged as promising carriers for ocular drug delivery. Conventional hydrogels can be classified as natural, such as alginate, chitosan, and hyaluronic acid, or synthetic, such as poly(ethylene glycol) (PEG), polyvinyl alcohol (PVA), and polyacrylamide [7,8,9,10,11].

The development of hydrogel-based ocular drug delivery has progressed from relatively simple inserts and contact lens-related concepts to multifunctional and responsive systems. Among these advances, in situ gelling systems have attracted particular attention because they can prolong ocular residence after administration. Subsequent innovations in drug-loaded contact lenses, diffusion-barrier strategies, sustained anti-VEGF delivery, smart responsive hydrogels, and tissue-engineering-related applications have further broadened the scope of this field, reflecting a shift toward more precise and multifunctional therapeutic platforms [12,13,14,15,16].

Bibliometrics is a research methodology that applies statistical and mathematical approaches to quantitatively analyze scientific publications, thereby revealing the developmental trajectories, knowledge architecture, research hotspots, and emerging frontiers within a given field. Commonly employed bibliometric tools include CiteSpace, VOSviewer, and the Bibliometrix R package. Although narrative reviews have summarized progress in hydrogel-based ocular drug delivery [12,13], and bibliometric studies have examined the broader field of ocular drug delivery [17], hydrogel-based ocular drug delivery as a distinct interdisciplinary research topic has not yet been systematically mapped in a focused manner. A dedicated bibliometric evaluation is therefore needed to clarify its long-term evolution, collaborative landscape, knowledge base, and hotspot shifts.

Accordingly, the present study conducted a bibliometric and visualized analysis of hydrogel-based ocular drug delivery research from 1991 to 2025. The aims were to characterize publication trends, map contributions and collaboration networks among countries, institutions, authors, and journals, and identify the major research hotspots and emerging directions in this field. These findings are expected to provide a clearer understanding of the field’s development and offer useful references for future research and translational innovation.

## 2. Materials and Methods

### 2.1. Data Source and Search Strategy

Bibliographic records were retrieved from the Web of Science Core Collection (WoSCC) on 25 August 2025, covering the period from database inception to the search date. WoSCC provides strict indexing, wide multidisciplinary coverage, and uniform citation metadata, which make it a reliable source for bibliometric studies.

The search strategy was structured around three conceptual domains: hydrogel-based materials, drug delivery, and ophthalmic applications. A topic search (TS) was performed using three conceptual domains: hydrogel-based materials, drug delivery, and ophthalmic applications. Only English-language articles and reviews were included, whereas Proceedings Papers, Early Access records, Retracted Publications, and Book Chapters were excluded. The final search query was as follows: TS = (hydrogel*) AND TS = (delivery) AND TS = (ophthal* OR ocular* OR eye*) AND LA = (English) AND DT = (Article OR Review) NOT DT = (“Proceedings Paper” OR “Early Access” OR “Retracted Publication” OR “Book Chapter”). The term “delivery” was intentionally retained to maximize search sensitivity, followed by manual screening to improve specificity.

### 2.2. Study Selection, Data Export, and Data Cleaning

All records were exported from WoSCC in Plain Text format with “Full Record and Cited References”. A total of 1678 records were identified, and one duplicate was removed. The remaining records were screened manually by title and abstract. Studies were included when hydrogels were the primary material and ophthalmic drug delivery was the main focus. Studies were excluded when hydrogels were mentioned only incidentally, when drug delivery was not the primary focus, or when the application was non-ophthalmic. Screening was conducted independently by two authors, and disagreements were resolved by a third author. After screening, 323 records were excluded, leaving 1354 publications for bibliometric analysis, including 991 articles and 363 reviews.

**Figure 1:**
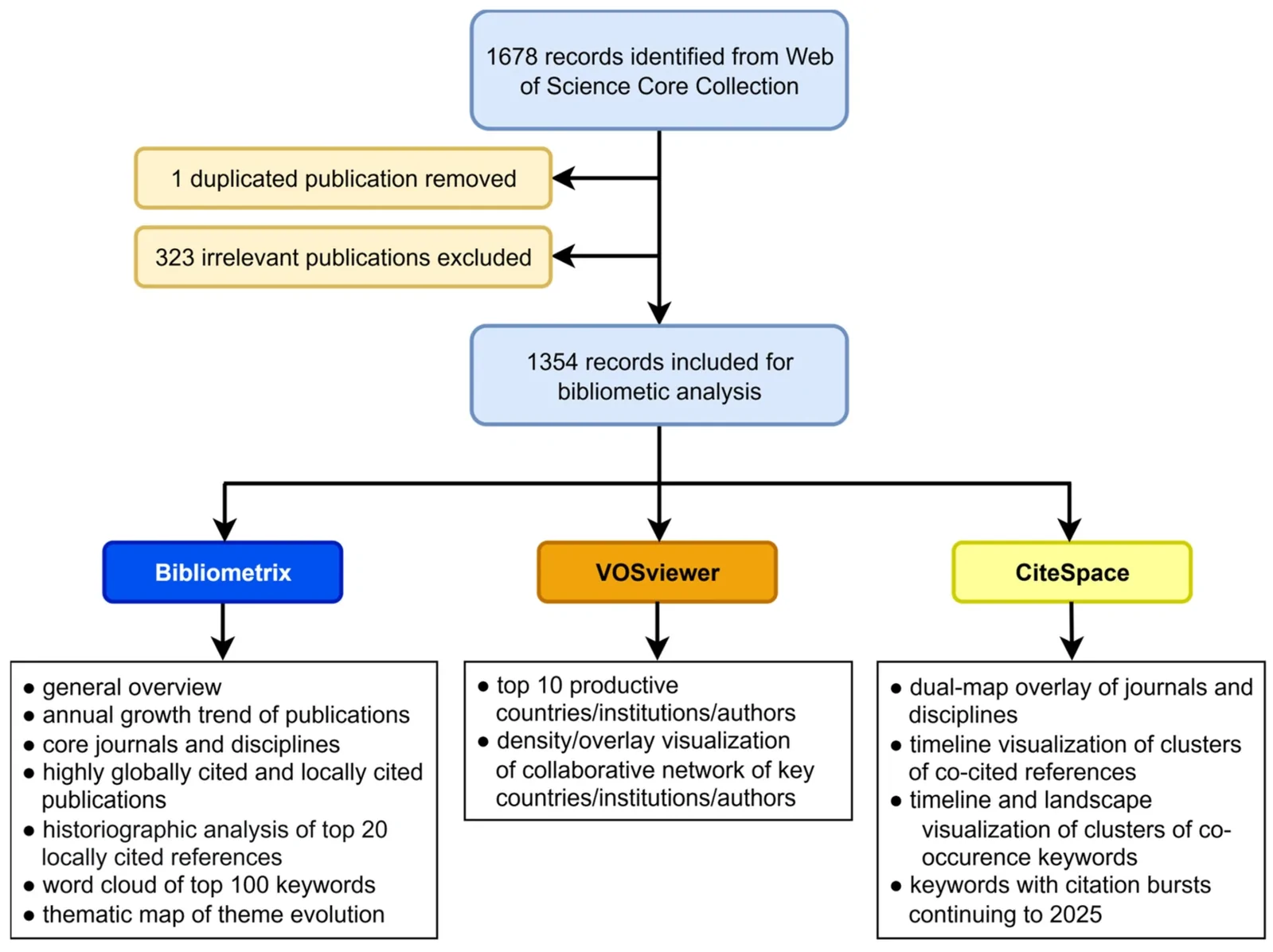
Flowchart of the data preparation and bibliometric analysis process.

Before bibliometric analysis, the exported records were further checked for duplication, missing information, and formatting consistency. Authors’ Keywords (DE) and Keywords Plus (ID) were extracted separately and were also combined to generate an integrated keyword dataset. Keyword standardization was performed through a structured procedure that included the following: (1) merging synonyms and closely related expressions; (2) harmonizing singular and plural forms; (3) standardizing spelling, capitalization, and hyphenation; (4) unifying abbreviations with their corresponding full terms; and (5) removing generic terms that provided limited value for thematic interpretation. The standardized terms were used only for keyword-based bibliometric analyses and did not alter the original WoSCC records, cited references, or publication titles. The overall study selection and data-processing workflow is presented in Figure 1.

### 2.3. Bibliometric Analysis, Visualization, and Indicators

The cleaned dataset was analyzed using the Bibliometrix R package (version 5.2.1), VOSviewer (version 1.6.20), and CiteSpace (version 6.1.R3). These tools were used complementarily to balance descriptive analysis, network visualization, and identification of emerging themes.

Bibliometrix (version 5.2.1) was used to analyze annual publication output, core journals, citation structures, historiographic relationships, word clouds, and thematic evolution.

VOSviewer (version 1.6.20) was used to construct co-authorship networks of countries/regions, institutions, and authors. The minimum publication thresholds were set at 20 publications for countries/regions, 10 for institutions, and 10 for authors. Minimum publication thresholds of 20 (countries/regions) and 10 (institutions and authors) were selected to retain active contributors and preserve network readability and interpretability.

CiteSpace (version 6.1.R3) was used to generate the dual-map overlay and to conduct keyword co-occurrence, reference co-citation, and citation burst analyses. A g-index with k = 25 was used to select nodes within each time slice for the keyword co-occurrence and reference co-citation analyses, retaining a broad range of influential nodes without creating overly dense or uninterpretable networks. Only keywords with burst periods extending to 2025 were retained to highlight current research frontiers.

The main bibliometric indicators included annual publication output, compound annual growth rate (CAGR), total citations, average citations per publication, local citation score, and total link strength. Annual publication output represented yearly research productivity, whereas CAGR reflected the overall rate of publication growth during the study period. Total citations and average citations per publication were used to describe citation impact within WoSCC. The local citation score indicated the number of citations received from publications included in the analyzed dataset. Total link strength, as calculated by VOSviewer, represented the overall strength of co-authorship relationships between a given node and the other nodes in the network.

## 3. Results

### 3.1. General Information

Following database retrieval and manual relevance screening, 1354 publications (991 articles and 363 reviews) published between 1991 and 2025 were included in the bibliometric analysis. These publications were distributed across 320 journals and involved 5744 authors from 1511 institutions in 70 countries/regions. International co-authorship accounted for 23.56% of the publications. The dataset accumulated 56,230 total citations, with an average of 41.53 citations per publication. Among all publications, 32 (2.36%) received more than 200 citations, and 122 (9.01%) received more than 100 citations.

**Figure 2:**
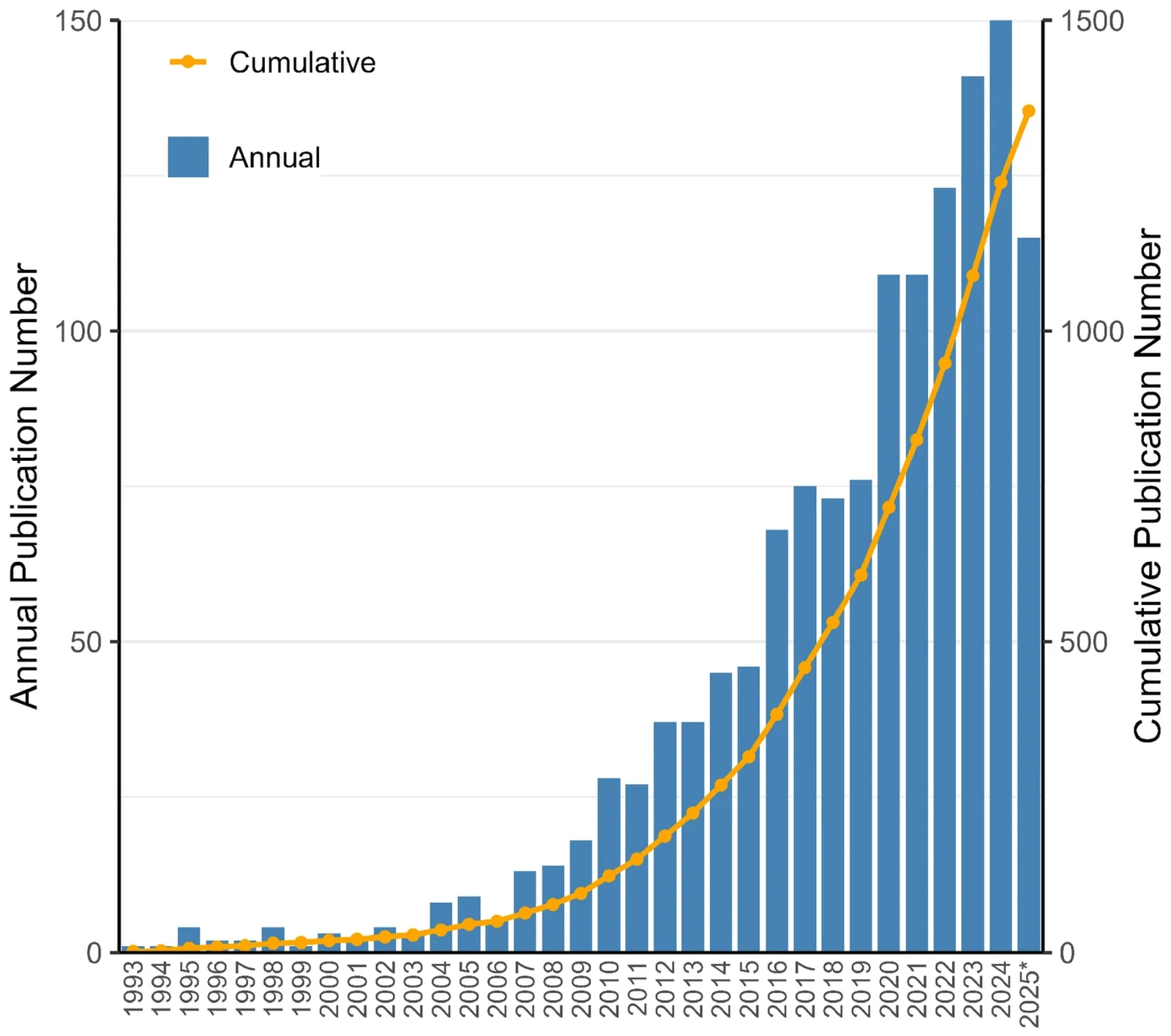
Annual and cumulative publication number of studies in hydrogel-based ocular drug delivery. *: as of 25 August 2025.

As shown in Figure 2, publication output progressed through three stages. From 1991 to 2007, the field remained in an early exploratory stage with limited annual output, which first exceeded 10 publications in 2007. From 2008 to 2019, output increased steadily, and 95.35% of all publications were published during or after 2008. Since 2020, the field has entered a rapid expansion stage, with annual output exceeding 100 publications for the first time in 2020. Publications indexed from 2020 to 25 August 2025 accounted for 55.17% of the total dataset. The overall CAGR from 1991 to 2025 was 14.98%, although this value mainly reflects long-term growth rather than stage-specific variation. Because data collection ended on 25 August 2025, the publication count for 2025 should be considered preliminary.

### 3.2. Contributions of Countries/Regions, Institutions, and Authors

Among the top 10 most productive countries/regions, four were from Europe, four from Asia, and two from North America (Table 1). China ranked first in publication output (390 publications), followed by the USA (274) and India (166), together contributing 61.3% of the total publications. In terms of citations, both China and the USA received more than 10,000 citations. However, despite having the highest publication output, China showed a lower average citation rate per paper (31.4) than both the overall dataset average (41.53) and the USA (64.2).

Among the top 10 most productive institutions, four were from China, and one was from the USA (Table 2). The University of Florida ranked first in publication output (55 publications) and also had the highest citation count (4317), making it the only institution with more than 4000 citations. Among the top 10 most productive authors (Table 3), three were affiliated with institutions in the USA, one in China, and one in India, while the others were from different countries. Chauhan, Anuj, was the most productive author, with publications in this field dating back to 2004.

**Figure 3:**
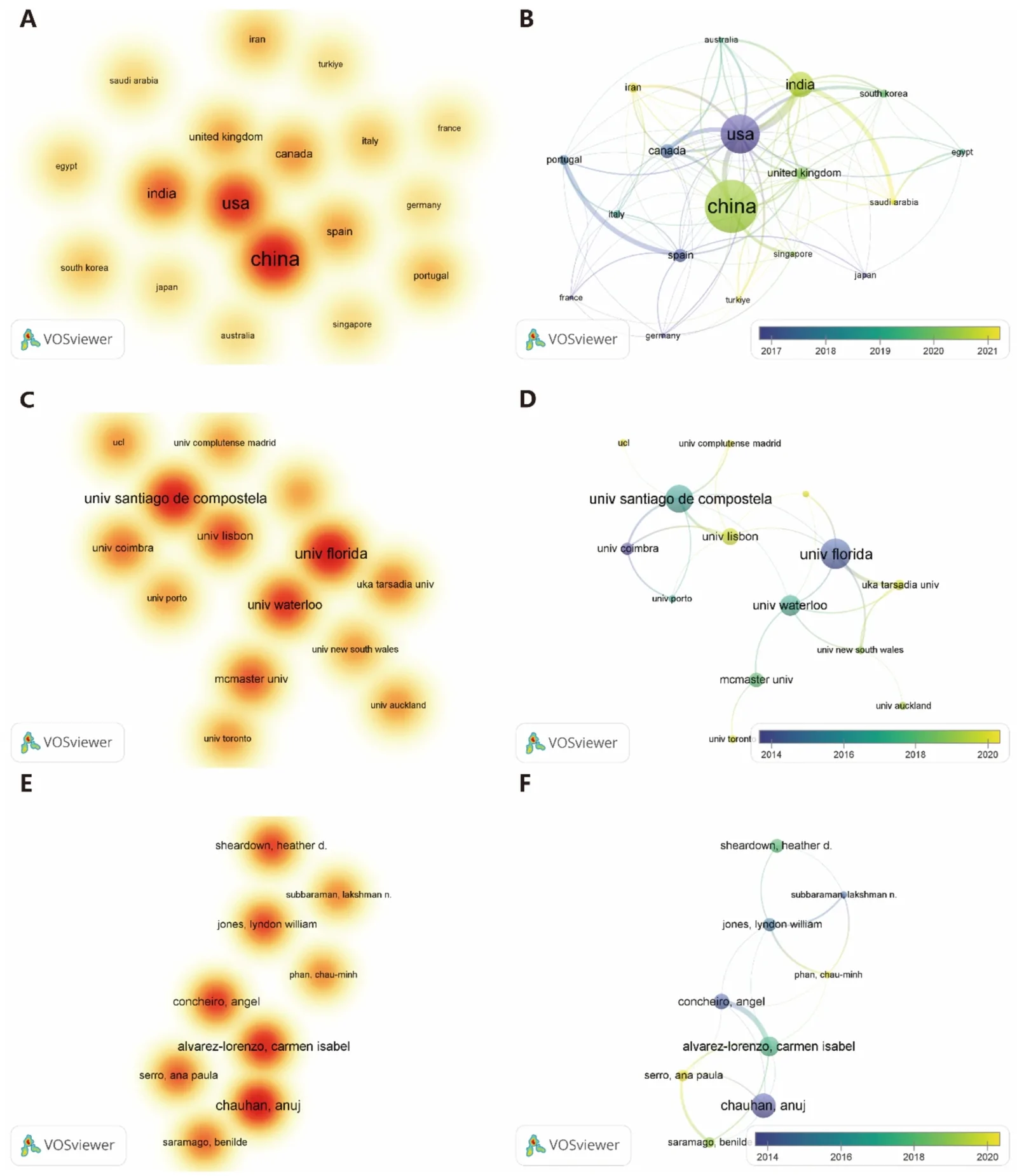
Visualization of co-authorship networks in hydrogel-based ocular drug delivery research by VOSviewer (version 1.6.20). (**A**) Density. (**B**) Overlay visualizations of country/region networks. (**C**) Density. (**D**) Overlay visualizations of institutional networks. (**E**) Density. (**F**) Overlay visualizations of author networks. In density views (**A**,**C**,**E**), node color intensity indicates publication count. In overlay views (**B**,**D**,**F**), node size is proportional to publication count, node color from blue to yellow indicates average publication year from early to late, and link thickness reflects collaboration link strength. Thresholds: countries/regions ≥ 20 publications, institutions and authors ≥ 10 publications.

Figure 3 shows the collaboration networks of countries/regions, institutions, and authors. At the country level, relatively strong collaborative links were observed between the USA and India (25), the USA and China (18), the USA and Canada (15), Spain and Portugal (17), and India and Saudi Arabia (16). Among the three most productive countries, the USA (TLS = 114) and India (TLS = 83) showed broader collaboration than China (TLS = 67). Overlay visualization suggested that the USA, Spain, Portugal, and Canada were earlier contributors, whereas China, India, Iran, and Saudi Arabia became more active more recently. At the institutional level, major collaborative clusters were centered on the University of Florida, the University of Santiago de Compostela, and the University of Waterloo. At the author level, the closest collaboration was observed between Alvarez-Lorenzo, Carmen Isabel, and Concheiro-Nine, Angel (link strength = 21). Although Chauhan, Anuj, had the highest publication output, collaboration strength in the displayed network remained relatively limited (TLS = 6).

### 3.3. Core Journals and Disciplines

The 1354 publications were distributed across 320 journals. The top 10 most productive journals, with three tied for ninth place (11 journals in total), published 437 papers, accounting for 32.3% of the dataset (Table 4). All were ranked in the 2024 Journal Citation Reports as Quartile 1 (Q1) journals, with Journal Impact Factors (JIFs) above 4.0, including seven above 5.0. *International Journal of Pharmaceutics* published the most papers (80 publications), followed by *Journal of Controlled Release* (54) and *Pharmaceutics* (51).

Based on the WoSCC WC field, these publications covered 59 subject categories. The three leading categories were Pharmacology and Pharmacy (541 publications, 39.96%), Polymer Science (232, 17.13%), and Materials Science, Biomaterials (222, 16.40%).

**Figure 4:**
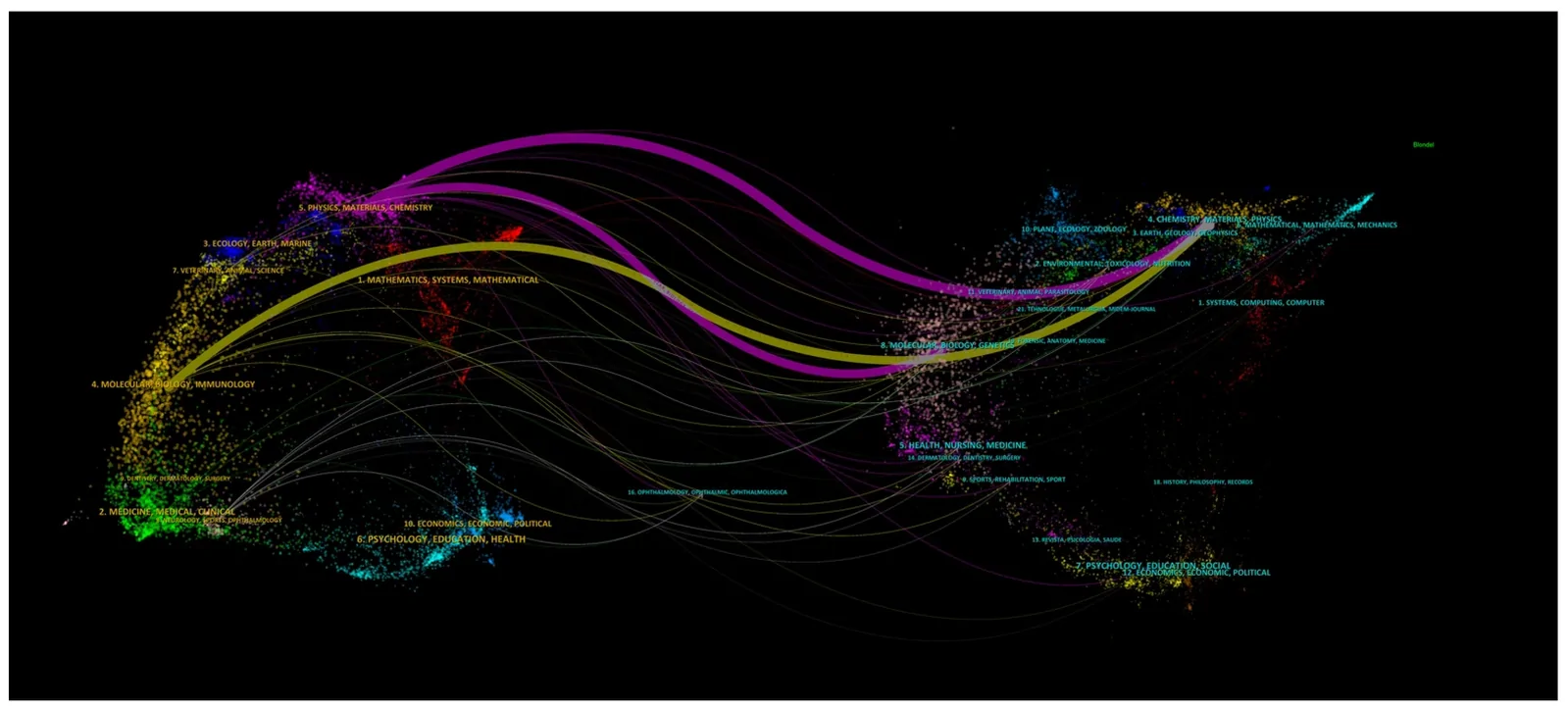
Dual-map overlay of citing and cited publications in hydrogel-based ocular drug delivery research by CiteSpace (version 6.1.R3). The labels on the left side represent the disciplines of the citing journals, and labels on the right side represent the disciplines of the cited journals. Colored curves illustrate the citation trajectories standardized using the *z*-score function.

A dual-map overlay of journals was generated to illustrate the citation relationships between journals and disciplines (Figure 4). Three main citation trajectories were identified: (1) “Molecular Biology, Immunology” journals mainly cited “Chemistry, Materials, Physics” journals; (2) “Physics, Materials, Chemistry” journals mainly cited journals in “Chemistry, Materials, Physics” and “Molecular Biology, Genetics”; and (3) repeated citations within “Chemistry, Materials, Physics” indicated continued emphasis on fundamental hydrogel materials research.

### 3.4. Research Topics and Hotspots

#### 3.4.1. Most Cited Publications

The most globally cited publications in the retrieved publications were predominantly reviews (17/20), indicating that broad academic influence in this field has been driven largely by review-based literature (Table 5). The most globally cited study was the 2004 review by Ruel-Gariépy E et al. [18], published in European Journal of Pharmaceutics and Biopharmaceutics, which received 1085 citations. Other highly cited papers also mainly addressed general ocular drug delivery, hydrogels, hyaluronic acid, and related biomaterial strategies.

**Figure 5:**
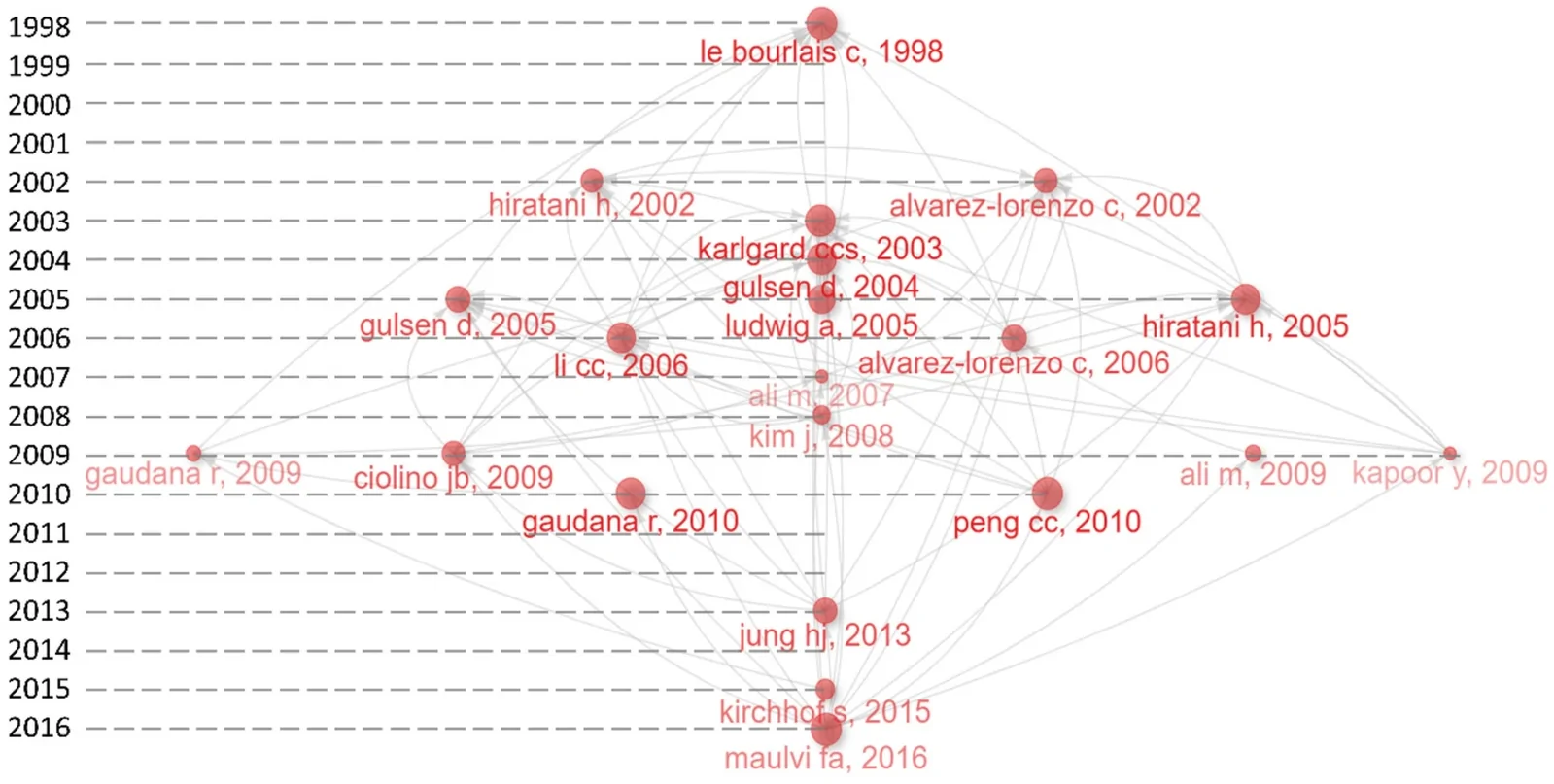
Historiographic map of the top 20 publications (with a tie at the 20th position, resulting in 21 papers) [14, 19, 20, 21, 22, 23,24,25,26,27,28,29,30,31,32,33,34,35,36,37,38] with the highest local citation counts (LCS) in hydrogel-based ocular drug delivery research by Bibliometrix R package (version 5.2.1). Each node represents a highly cited publication, with node size proportional to its local citation count. Arrows indicate directional citation relationships between the publications.

By contrast, the local citation profile more directly revealed the field’s knowledge base. In the present study, the top 20 most locally cited publications are shown in a historiography (Figure 5). These key locally cited studies were published between 1998 and 2016 and focused primarily on contact lens-based ocular delivery, silicone-hydrogel systems, molecularly imprinted hydrogels, and sustained timolol release. The most locally cited paper was an article published by Peng CC et al. in Biomaterials in 2010 on Vitamin E diffusion barriers in silicone-hydrogel contact lenses, with 117 local citations.

Only six studies (Le Bourlais C, 1998 [19], Prog Retin Eye Res; Gaudana R, 2010 [36], AAPS J; Gulsen D, 2004 [23], Invest Ophth Vis Sci; Hiratani H, 2005 [24], Biomaterials; Ludwig A, 2005 [25], Adv Drug Deliver Rev; Gaudana R, 2009 [33], Pharm Res-Dordr) overlapped between the global- and local-citation lists, indicating that broadly influential publications were not fully identical to those underpinning the field’s intellectual foundation. Overall, global citation counts highlighted literature with wider disciplinary visibility, whereas local citations more clearly identified the core references that shaped the development of hydrogel-based ocular drug delivery research.

#### 3.4.2. Most Locally Cited References and Co-Citation Analysis

**Figure 6:**
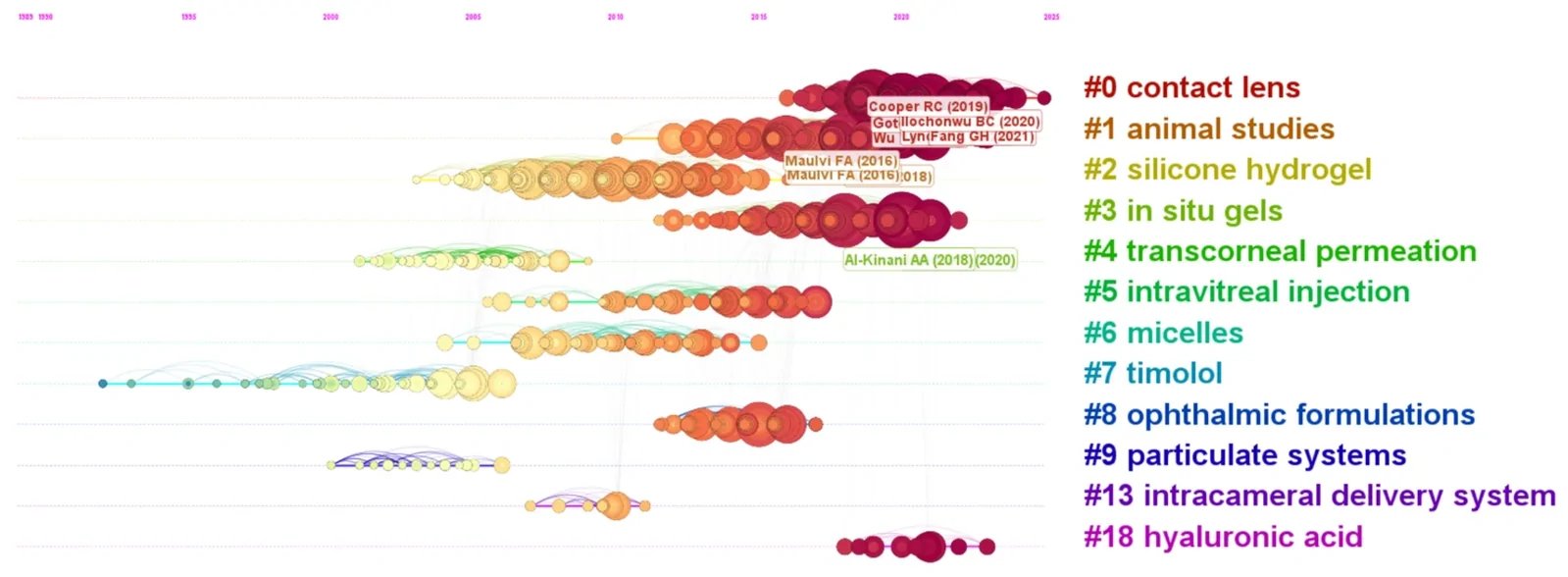
Timeline visualization of co-cited references in hydrogel-based ocular drug delivery research by CiteSpace (version 6.1.R3). Node size is proportional to citation frequency.

Among the 60,153 references cited by the 1354 publications, the top 10 most locally cited references are listed in Table 6. These references were identical to the top 10 publications with the highest local citation counts reported above, indicating that the field’s most influential publications also constituted its principal knowledge base. Beyond the top 10, however, the rankings no longer overlapped completely, suggesting a broader and more diverse intellectual foundation. These highly cited references were concentrated on ocular drug delivery systems, therapeutic contact lenses, and hydrogel-based drug-release strategies, which together formed the core knowledge base of the field. Figure 6 further complements these findings by illustrating key co-citation clusters and their temporal trends, delineating the knowledge structure and developmental trajectory of this research field.

#### 3.4.3. Keyword Frequency, Co-Occurrence, Thematic Map, and Burst Detection

**Figure 7:**
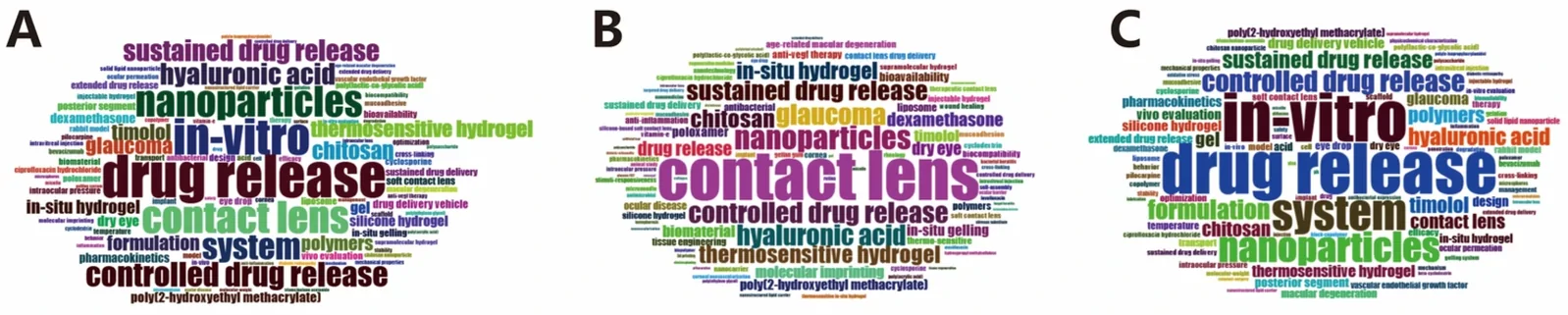
Word clouds of the top 100 high-frequency keywords in hydrogel-based ocular drug delivery research by the Bibliometrix R package (version 5.2.1): (**A**) combined keywords; (**B**) authors’ keywords; (**C**) keywords plus.

A total of 2894 authors’ keywords and 2699 Keywords Plus were extracted from the 1354 included publications. After synonym merging, the top 100 frequent keywords were visualized in word clouds for total keywords, authors’ keywords, and Keywords Plus, respectively (Figure 7). Excluding search-strategy terms and their synonyms, keywords with a frequency exceeding 100 included drug release, in vitro, contact lens, nanoparticles, system, controlled drug release, hyaluronic acid, sustained drug release, chitosan, glaucoma, thermosensitive hydrogel, timolol, formulation, polymers, and in situ hydrogel.

**Figure 8:**
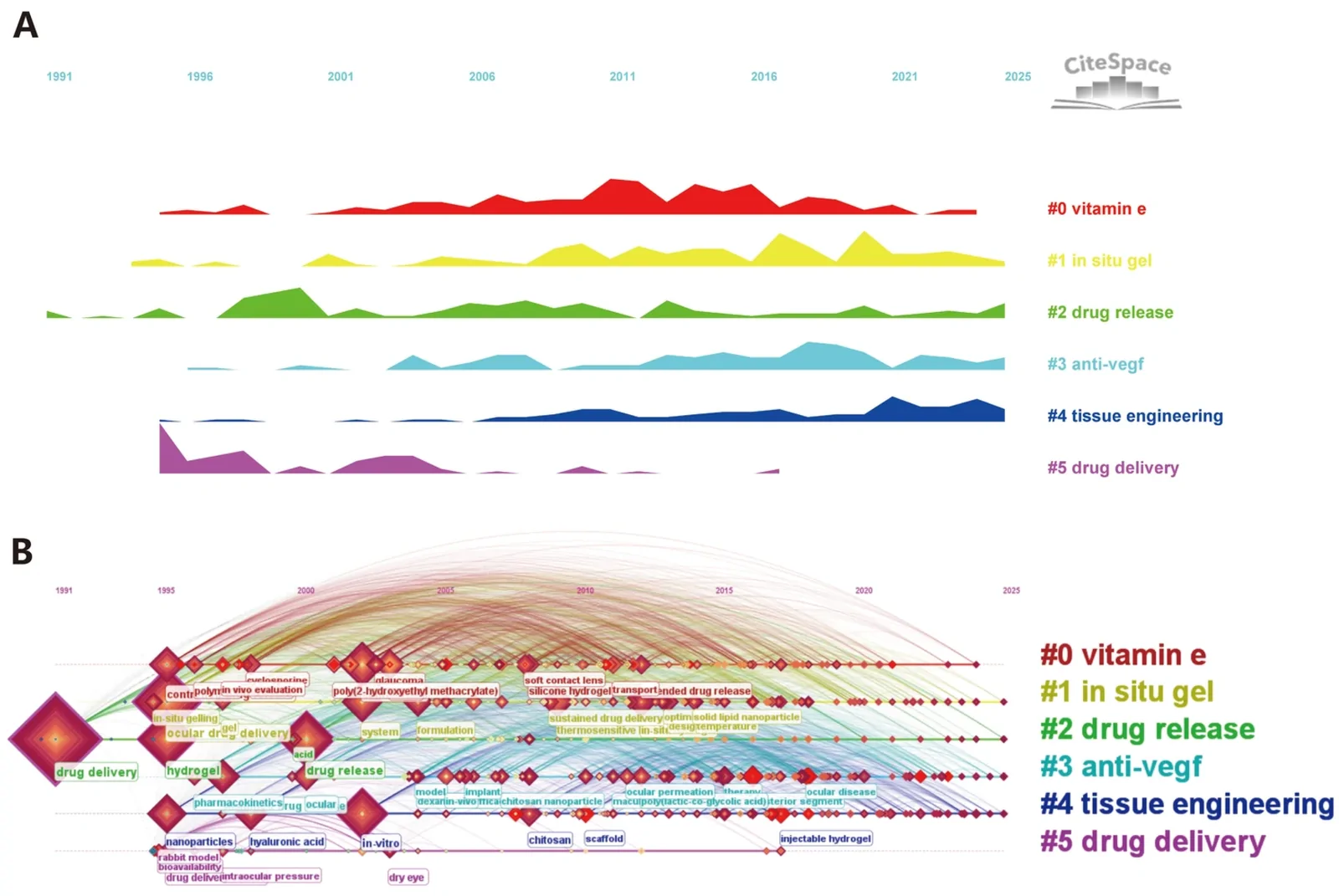
(**A**) The landscape view and (**B**) timeline view of the co-occurring analysis of significant clusters of keywords by CiteSpace (version 6.1.R3). Node size is proportional to keyword occurrence frequency in the timeline view.

The landscape view and timeline view of keyword co-occurrence (Figure 8) illustrated the temporal evolution of research topics. Early studies were mainly characterized by the clusters “drug release” and “drug delivery”, whereas “vitamin E” became prominent in the 2010s. More recently, “in situ gel”, “anti-VEGF”, and “tissue engineering” have attracted increasing attention, indicating continued expansion of the field toward advanced formulations and broader therapeutic applications.

**Figure 9:**
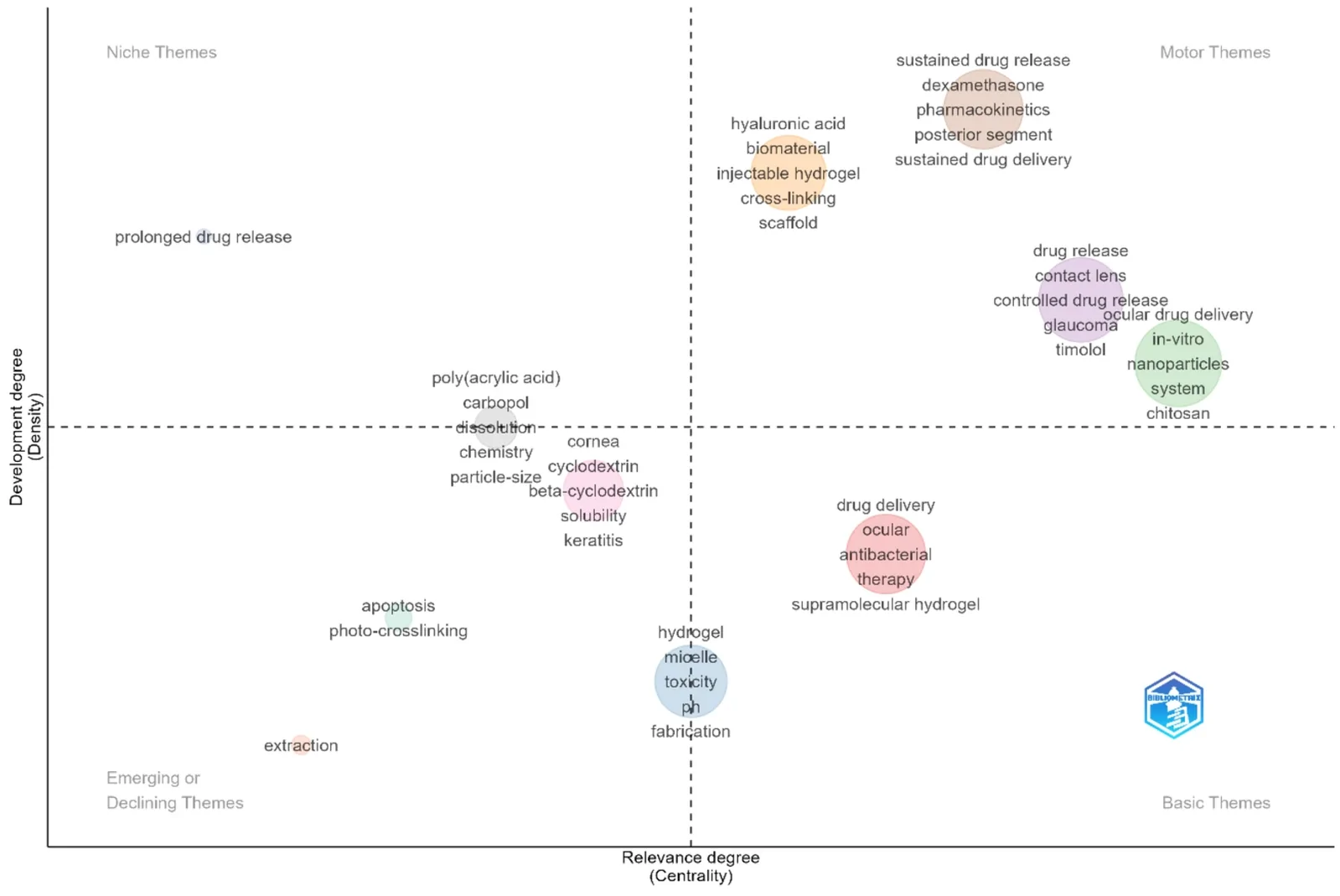
The thematic map of the combined keywords by the Bibliometrix R package (version 5.2.1). The size of the circles is proportional to the total occurrence frequency of keywords within each keyword cluster.

In the thematic map (Figure 9), keywords with a frequency greater than three were grouped into 11 clusters and distributed across four quadrants according to centrality and density. Among the motor themes, the cluster labeled with “ocular drug delivery”, “in vitro”, “nanoparticles”, “system”, and “chitosan” showed the highest centrality, whereas the cluster labeled with “sustained drug release”, “dexamethasone”, “pharmacokinetics”, “posterior segment”, and “sustained drug delivery” showed the highest density. The cluster labeled “prolonged drug release” was located in the niche-theme quadrant, indicating a specialized but relatively peripheral topic. By contrast, the cluster labeled with “drug delivery”, “ocular”, “antibacterial”, “therapy”, and “supramolecular hydrogel” was positioned in the basic-theme quadrant, suggesting a foundational but still underdeveloped area. The cluster labeled “extraction” showed the lowest centrality and density, indicating a marginal and weakly developed topic.

**Figure 10:**
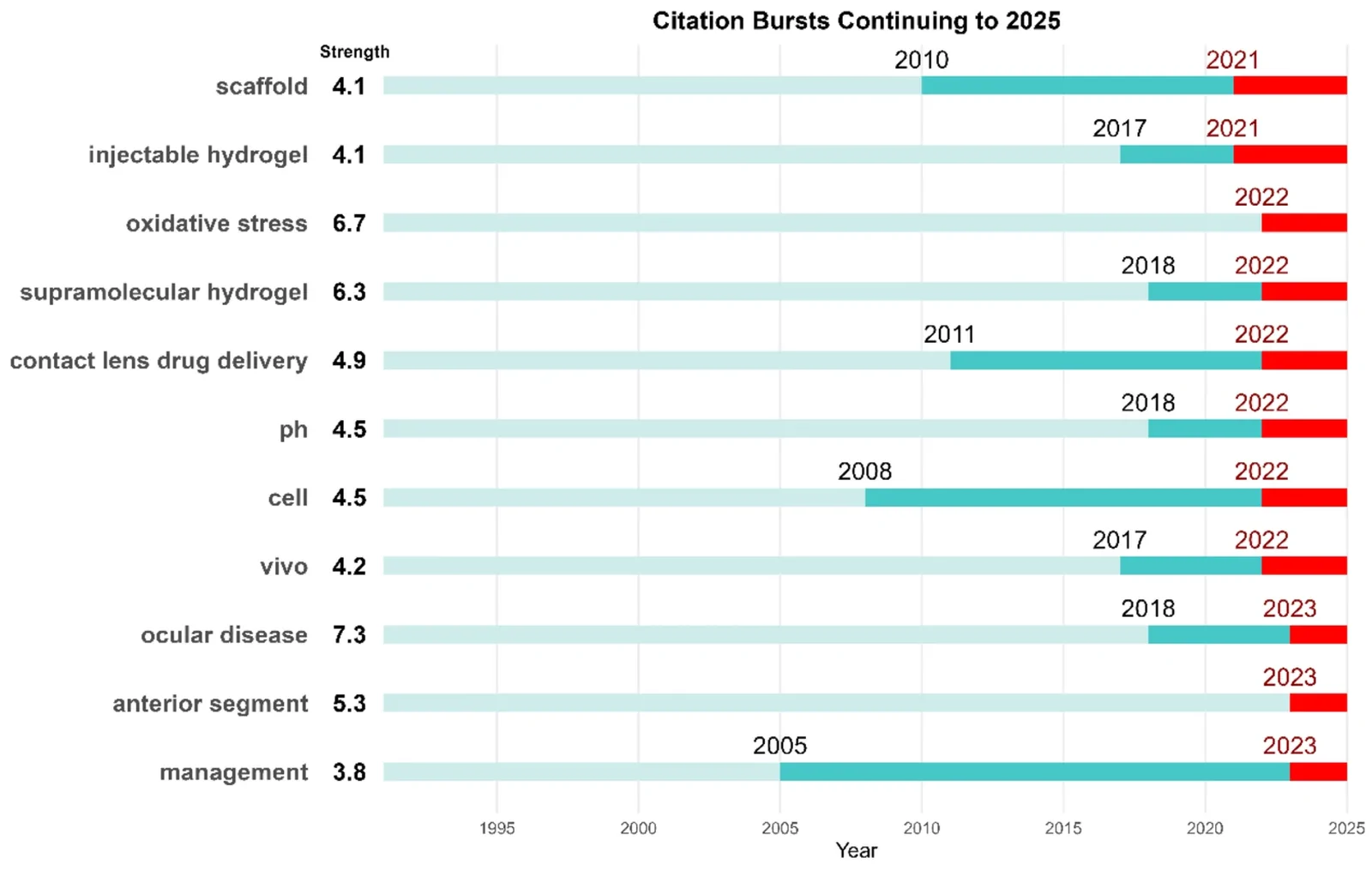
Keywords with citation bursts continuing to 2025.

Burst detection analysis (Figure 10) identified 11 keywords with ongoing citation bursts through 2025, all of which began after 2020. Among them, the strongest bursts were observed for “ocular disease” (strength = 7.3), “oxidative stress” (strength = 6.7), and “supramolecular hydrogel” (strength = 6.3). These burst terms suggest that recent research attention has shifted toward disease-focused investigation, mechanistic exploration, and emerging hydrogel materials.

#### 3.4.4. Disease- and Ocular-Segment-Related Thematic Patterns

Based on the thematic evolution of co-occurring keywords (Figure 8 and Figure 9), keywords with ongoing citation bursts (Figure 10), and representative highly cited publications (Table 5; Figure 5), hydrogel-based ocular drug delivery research can also be contextualized within a disease- and ocular-segment-related perspective.

Anterior segment- and surface-oriented themes were more frequently associated with keywords such as “contact lens”, “glaucoma”, “timolol”, “in situ hydrogel”, and “thermosensitive hydrogel”, together with persistent emphasis on “drug release” and “controlled drug release” among the most frequent terms (Figure 8 and Figure 9). These patterns suggest sustained interest in noninvasive or topical hydrogel-based strategies relevant to anterior ocular therapy.

Posterior segment-oriented themes were more closely linked to later-emerging and highly developed terms such as “anti-VEGF”, “posterior segment”, “sustained drug release”, “sustained drug delivery”, “dexamethasone”, and “pharmacokinetics” (Figure 8 and Figure 9). This interpretation was further supported by the burst term “ocular disease”, indicating increasing recent attention to disease-focused therapeutic applications (Figure 10). Overall, these patterns suggest that the field has gradually expanded from early emphasis on drug-release-centered and surface-oriented systems toward more disease-focused and posterior segment-related delivery platforms.

## 4. Discussion

### 4.1. General Overview

Research on hydrogel-based ocular drug delivery has expanded substantially over the past three decades, indicating sustained growth of interest in this field rather than a short-lived concentration on a single technology. The knowledge base and collaboration structure further suggest that this area has gradually evolved into an internationally active and interdisciplinary domain, with China and the USA serving as major contributors and several productive teams shaping its development. More importantly, the field has progressed from early emphasis on material properties and release control toward clinically oriented applications, particularly those addressing prolonged ocular residence, posterior segment delivery, and disease-specific therapeutic needs.

The accelerated growth after 2007–2008 likely reflects several converging drivers. One plausible explanation is the expansion of the anti-VEGF treatment era, which substantially increased demand for sustained ocular delivery strategies, particularly for posterior segment disease [39,40]. This growth may also be associated with the maturation of biomaterials, nanotechnology, and stimuli-responsive material design, which broadened the functional scope of hydrogel systems and made them more adaptable to ophthalmic applications [4,41]. In parallel, the rapid rise in research output in Asia, especially in China, likely contributed to the marked increase in global publication volume. The persistent need for long-acting ocular therapies, driven by poor bioavailability of conventional formulations and the treatment burden of repeated intraocular administration, probably provided an additional and durable impetus for this expansion [4,42].

### 4.2. Research Themes and Technological Landscapes

The bibliometric findings indicate that hydrogel-based ocular drug delivery has undergone substantial thematic broadening over time. Earlier studies were more strongly centered on material selection, hydrogel properties, drug loading, and basic release control [35,43,44,45,46], whereas subsequent research increasingly incorporated application-oriented themes, including controlled and sustained drug delivery, disease-specific requirements, posterior-segment administration, pharmacokinetics, and responsive hydrogel systems [41]. This development is also reflected in the distinction between globally and locally cited publications. The most globally cited papers were predominantly broad reviews addressing ocular drug delivery, hydrogels, hyaluronic acid, and related biomaterials, suggesting that the international visibility of the field has been shaped largely by conceptual and platform-level syntheses. By contrast, the local citation network more clearly represented its specialized intellectual base, particularly in contact-lens-assisted delivery, silicone-hydrogel systems [35,43,47,48,49,50,51,52,53,54,55,56], molecularly imprinted hydrogels [47,57], and sustained timolol release.

The technological landscape encompasses several platforms adapted to different administration routes and therapeutic objectives. Contact-lens-based systems remain prominent in anterior-segment delivery, where they are investigated to prolong precorneal residence and regulate drug release [57,58,59]. The continued visibility of terms such as “contact lens”, “timolol”, “drug release”, and “controlled drug release” reflects sustained activity in this area. Topical in situ gelling systems, which typically rely on temperature, pH, or ionic strength to trigger sol-to-gel transition, constitute another established branch [60,61,62,63,64,65,66,67,68,69,70], combining the convenience of administration as a liquid formulation with increased local retention following gel formation [71,72]. Advanced stimuli-responsive hydrogels further extend this approach by enabling changes in rheological or structural properties, as well as on-demand drug release under specific pathological conditions [41].

Injectable and depot-forming hydrogels have become increasingly visible in association with sustained intraocular delivery, posterior-segment therapy, pharmacokinetics, and anti-VEGF agents [73,74,75]. Their development reflects the need for prolonged drug exposure in anatomical compartments that are difficult to reach through topical administration. Depending on their composition and gelation mechanism, these systems may allow minimally invasive administration followed by local depot formation and controlled release. Their design requirements differ from those of contact lenses and topical in situ gels, particularly with respect to injectability, gelation kinetics, depot stability, degradation, and compatibility with sensitive therapeutic agents.

Responsive, hybrid, and multifunctional systems have further diversified this technological landscape [41,45,76,77,78,79,80,81,82,83,84,85]. The integration of hydrogel matrices with affinity interactions, molecular imprinting, particulate carriers, or environment-sensitive components may provide additional control over drug loading, localization, and release. Collectively, the bibliometric patterns reveal the parallel development of surface-retentive, contact-lens-based, in situ gelling, and injectable depot-forming systems, with their relative research prominence shaped by the targeted ocular compartment, administration route, drug properties, and intended duration of therapy.

### 4.3. Disease- and Ocular-Segment-Specific Implications

The disease- and ocular-segment-related thematic patterns identified in this study provide an additional perspective on the diversification of hydrogel-based ocular drug delivery. Keywords such as “contact lens”, “glaucoma”, “timolol”, “in situ hydrogel”, and “thermosensitive hydrogel” were mainly associated with anterior-segment and ocular-surface delivery. In contrast, “anti-VEGF”, “posterior segment”, “sustained drug delivery”, “dexamethasone”, and “pharmacokinetics” were more closely related to intraocular and posterior-segment applications. These differences reflect segment-specific anatomical barriers, administration routes, clearance mechanisms, and therapeutic-duration requirements [86,87,88].

Anterior-segment research emphasizes topical, contact-lens-assisted, and in situ gelling systems. The prominence of “glaucoma” and “timolol” indicates sustained interest in improving topical treatment for chronic ocular diseases. Contact-lens-assisted systems can prolong drug release at the ocular surface [57,58,89], whereas in situ hydrogels may enhance local retention through physiologically triggered gelation [71,90,91]. Nevertheless, prolonged residence alone does not establish improved bioavailability, which also depends on drug release, corneal permeability, tissue distribution, and local clearance.

Posterior-segment themes reflect growing interest in long-acting intraocular delivery, sustained intraocular exposure, pharmacokinetics, and anti-angiogenic or anti-inflammatory therapy. Injectable in situ forming hydrogels may function as local depots, but their performance depends on injectability, gelation, release kinetics, depot persistence, tissue distribution, and ocular pharmacokinetics [73,75,92]. The emergence of “anti-VEGF” further highlights sustained delivery of biologics, for which molecular stability and biological activity must be preserved throughout formulation and release [39,40,42,76,77,78,79,80,81,93,94,95,96,97,98]. “Dexamethasone” similarly reflects interest in sustained anti-inflammatory therapy, although it is not specific to a single ocular segment [99].

### 4.4. Pharmaceutical Significance of the Bibliometric Trends

From a pharmaceutical perspective, hydrogels have evolved from simple viscosity- and retention-enhancing materials into route-, disease-, and payload-adapted delivery systems [100]. Current strategies include in situ gelation, stimuli-responsive hydrogels, functionally engineered soft contact lenses, injectable depot formation, and integration with particulate carriers [12,41,83,101,102,103,104,105,106,107,108,109,110,111,112,113,114,115,116,117,118]. This evolution reflects a shift from material-centered screening toward controlled release, improved ocular exposure, and prolonged therapeutic effects [14,16,40,41,119].

Formulation requirements differ between ocular segments [12]. Anterior-segment systems require appropriate rheology, tear-film compatibility, ocular retention, and tolerability. Drug-delivering contact lenses must also maintain transparency, oxygen permeability, mechanical properties, drug loading, and reproducible release [120,121]. Topical in situ gels should remain easy to administer and undergo rapid but controlled gelation under ocular conditions [122,123]. By contrast, posterior-segment hydrogels are generally injectable or implantable depots [124]. Their key attributes include injectability, gelation time, depot stability, burst-release control, degradation, and intraocular biocompatibility [14,15,40,41,42,125].

The prominence of “controlled drug release” and “sustained drug release” emphasizes the importance of release mechanisms [126]. Release may be governed by diffusion, degradation, erosion, or drug-polymer interactions [100]. Hybrid hydrogels containing nanoparticles, liposomes, or polymeric micelles can provide multilevel release control but may increase the complexity of characterization, sterilization, manufacturing, and quality control [117].

The properties of the encapsulated therapeutic payload represent another critical formulation consideration. Biologics, including anti-VEGF agents, are susceptible to aggregation, adsorption, degradation, and loss of activity [127,128]. Their evaluation should therefore include protein integrity, potency, and biological activity rather than cumulative release alone [129]. Despite these advances, bibliometric prominence does not indicate clinical adoption [12]. Translation still depends on sterilization, storage stability, scalability, batch reproducibility, administration reliability, and long-term ocular safety [42,46,119].

### 4.5. Limitations

This bibliometric analysis has several limitations. First, the search was restricted to English articles and reviews in the WoSCC database, potentially omitting relevant publications from other databases or languages. Database coverage and indexing practices may also affect representativeness. Second, the search strategy required delivery-related terms, thereby potentially resulting in the omission of relevant studies that do not explicitly utilize the target term. Third, citation counts favor older papers, while recent high-impact studies may be underrepresented. High citations reflect academic visibility, not necessarily methodological quality, performance, or clinical relevance. Finally, the analysis depends solely on publications and citation metadata and excludes patents, clinical trials, marketed products, or regulatory data. Consequently, a direct assessment of commercialization, regulatory progress, or translational success is precluded. Any discussion of translational potential is an indirect inference from publication trends.

### 4.6. Future Perspectives

Future research will likely follow two complementary directions: patient-friendly anterior-segment delivery and long-acting posterior-segment therapy. For anterior-segment systems, contact lenses and topical in situ hydrogels should balance prolonged retention with comfort, visual performance, oxygen permeability, reproducible dosing, and ease of use [59,120,121]. Their pharmacokinetic and therapeutic benefits should be directly compared with conventional eye drops [71,90,91].

For posterior-segment delivery, injectable in situ forming hydrogels should combine sustained exposure with injectability, stability, biocompatibility, and preservation of biologic activity, particularly for anti-VEGF agents [16,40,41,42,73,75,77,78,92]. Hybrid systems incorporating nanoparticles or other carriers may improve drug protection and release control [117,118], but their added complexity must provide clear advantages [100].

Future development should also address sterilization, scale-up, storage stability, batch reproducibility, device compatibility, and long-term ocular safety [12,119,125]. Standardized testing and integration of publication, patent, clinical-trial, and regulatory data will help clarify the translational potential of these platforms [74,124].

## 5. Conclusions

This bibliometric analysis demonstrates the sustained growth and diversification of hydrogel-based ocular drug delivery over the past 35 years. Research has progressed from material design and basic release control toward disease- and segment-specific applications, including contact lenses and topical in situ gels for anterior-segment delivery and injectable, sustained-release systems for posterior-segment therapy. Anti-VEGF delivery and stimuli-responsive hydrogels have emerged as prominent research frontiers. Overall, the field is moving toward more precise, long-acting, and clinically relevant delivery strategies, although further progress will depend on formulation stability, ocular safety, manufacturability, and successful translation into clinical practice.

## Figures and Tables

**Table 1:** Top 10 countries by publication count in hydrogel-based ocular drug delivery.

Rank	Country	Documents	Citations per Paper	Citations	Production Year Start
1	People’s Republic of China	390	31.4	12,248	2001
2	USA	274	64.2	17,592	1993
3	India	166	33.5	5564	1995
4	Canada	86	41.6	3579	2003
5	Spain	78	43.7	3408	2002
6	UK	72	38.6	2780	1998
7	Portugal	57	38.2	2179	2007
7	Iran	57	23.8	1355	2012
9	South Korea	46	43.8	2014	2003
10	Italy	44	51.6	2271	1996

**Table 2:** Top 10 institutions by publication count in hydrogel-based ocular drug delivery.

Rank	Institution	Country	Documents	Citations	Production Year Start
1	University of Florida	USA	55	4317	1994
2	The University of Santiago de Compostela	Spain	49	2472	2002
3	Eye Hospital of Wenzhou Medical University	People’s Republic of China	47	1334	2014
4	University of Waterloo	Canada	35	1406	2003
5	Wenzhou Medical University	People’s Republic of China	34	1476	2013
6	Shenyang Pharmaceutical University	People’s Republic of China	33	1448	2008
7	University of Lisbon	Portugal	28	752	2014
8	McMaster University	Canada	24	390	2011
9	University of Coimbra	Portugal	19	1004	2007
10	Chang Gung University	People’s Republic of China	17	888	2010

**Table 3:** Top 10 authors by publication count in hydrogel-based ocular drug delivery.

Rank	Author	Institution	Country	Documents	Citations	Production Year Start
1	Chauhan, Anuj	Colorado School of Mines, University of Florida	USA	40	3179	2004
2	Li, Xingyi	Wenzhou Medical University	People’s Republic of China	33	1307	2013
3	Alvarez-Lorenzo, Carmen Isabel	The University of Santiago de Compostela	Spain	31	1364	2006
4	Concheiro-Nine, Angel	The University of Santiago de Compostela	Spain	24	1419	2002
5	Sheardown, Heather D.	McMaster University	Canada	21	344	2011
6	Jones, Lyndon William	University of Waterloo	Canada	20	799	2003
7	Serro, Ana Paula	University of Lisbon	Portugal	17	384	2016
8	Byrne, Mark Edward	Rowan University	USA	16	1029	2007
8	Kang-Mieler, Jennifer J.	Illinois Institute of Technology	USA	16	608	2011
9	Maulvi, Furqan A.	Uka Tarsadia University	India	15	987	2015
9	Saramago, Benilde	University of Lisbon	Portugal	15	405	2014

**Table 4:** Top 10 journals by publication count in hydrogel-based ocular drug delivery.

Rank	Journal	Publications	JIF (2024)	Category	JIF Quartile (2024)	Production Year Start
1	*INTERNATIONAL JOURNAL OF PHARMACEUTICS*	80	5.2	PHARMACOLOGY and PHARMACY	Q1	2003
2	*JOURNAL OF CONTROLLED RELEASE*	54	11.5	CHEMISTRY, MULTIDISCIPLINARY/PHARMACOLOGY and PHARMACY	Q1/Q1	1997
3	*PHARMACEUTICS*	51	5.5	PHARMACOLOGY and PHARMACY	Q1	2018
4	*INTERNATIONAL JOURNAL OF BIOLOGICAL MACROMOLECULES*	48	8.5	BIOCHEMISTRY and MOLECULAR BIOLOGY/CHEMISTRY, APPLIED/POLYMER SCIENCE	Q1/Q1/Q1	2012
5	*EUROPEAN JOURNAL OF PHARMACEUTICS AND BIOPHARMACEUTICS*	45	4.3	PHARMACOLOGY and PHARMACY	Q1	1996
6	*JOURNAL OF DRUG DELIVERY SCIENCE AND TECHNOLOGY*	41	4.9	PHARMACOLOGY and PHARMACY	Q1	2007
7	*GELS*	27	5.3	POLYMER SCIENCE	Q1	2020
8	*ACTA BIOMATERIALIA*	25	9.6	ENGINEERING, BIOMEDICAL/MATERIALS SCIENCE, BIOMATERIALS	Q1/Q1	2009
9	*BIOMATERIALS*	22	12.9	ENGINEERING, BIOMEDICAL/MATERIALS SCIENCE, BIOMATERIALS	Q1/Q1	2011
9	*CARBOHYDRATE POLYMERS*	22	12.5	CHEMISTRY, APPLIED/CHEMISTRY, ORGANIC/POLYMER SCIENCE	Q1/Q1/Q1	2001
9	*POLYMERS*	22	4.9	POLYMER SCIENCE	Q1	2014

**Table 5:** Top 20 publications by total global citations in hydrogel-based ocular drug delivery.

Rank	Title	First Author	Journal	Year	Type	Total Global Citations	TC per Year
1	In situ-forming hydrogels—review of temperature-sensitive systems	RUEL-GARIÉPY E	*EUR J PHARM BIOPHARM*	2004	Review	1085	49.32
2	Ocular drug delivery	GAUDANA R	*AAPS J*	2010	Review	947	59.19
3	Hyaluronic acid: a natural biopolymer with a broad range of biomedical and industrial applications	KOGAN G	*BIOTECHNOL LETT*	2007	Review	759	39.95
4	Nanosuspensions: a promising drug delivery strategy	PATRAVALE VB	*J PHARM PHARMACOL*	2004	Review	681	30.95
5	The use of mucoadhesive polymers in ocular drug delivery	LUDWIG A	*ADV DRUG DELIVER REV*	2005	Review	680	32.38
6	Chemical modifications of hyaluronic acid for the synthesis of derivatives for a broad range of biomedical applications	SCHANTÉ CE	*CARBOHYD POLYM*	2011	Review	557	37.13
7	Ophthalmic drug delivery systems—Recent advances	LE BOURLAIS C	*PROG RETIN EYE RES*	1998	Review	548	19.57
8	Cross-linked hyaluronic acid hydrogel films: new biomaterials for drug delivery	LUO Y	*J CONTROL RELEASE*	2000	Article	503	19.35
9	Self-Healing Injectable Hydrogels for Tissue Regeneration	BERTSCH P	*CHEM REV*	2023	Review	497	165.67
10	Target specific and long-acting delivery of protein, peptide, and nucleotide therapeutics using hyaluronic acid derivatives	OH EJ	*J CONTROL RELEASE*	2010	Review	470	29.38
11	Recent Perspectives in Ocular Drug Delivery	GAUDANA R	*PHARM RES-DORDR*	2009	Review	432	25.41
12	Emerging Role of Hydrogels in Drug Delivery Systems, Tissue Engineering and Wound Management	JACOB S	*PHARMACEUTICS*	2021	Review	304	60.80
13	Poloxamer Hydrogels for Biomedical Applications	RUSSO E	*PHARMACEUTICS*	2019	Review	300	42.86
14	Application of gellan gum in pharmacy and medicine	OSMALEK T	*INT J PHARMACEUT*	2014	Review	291	24.25
15	Advances and limitations of drug delivery systems formulated as eye drops	JUMELLE C	*J CONTROL RELEASE*	2020	Review	268	44.67
16	Penetration enhancers and ocular bioadhesives: Two new avenues for ophthalmic drug delivery	KAUR IP	*DRUG DEV IND PHARM*	2002	Review	245	10.21
17	Ocular release of timolol from molecularly imprinted soft contact lenses	HIRATANI H	*BIOMATERIALS*	2005	Article	245	11.67
18	Ophthalmic drug delivery through contact lenses	GULSEN D	*INVEST OPHTH VIS SCI*	2004	Article	244	11.09
19	Recent advances in ocular drug delivery	ACHOURI D	*DRUG DEV IND PHARM*	2013	Review	242	18.62
19	Hyaluronic Acid and Controlled Release: A Review	BAYER IS	*MOLECULES*	2020	Review	242	40.33

**Table 6:** Top 10 references by local citations in hydrogel-based ocular drug delivery.

Rank	Citations	Title	First Author	Journal	Publication Year	Type	DOI
1	117	Extended delivery of hydrophilic drugs from silicone-hydrogel contact lenses containing vitamin E diffusion barriers	PENG CC	*BIOMATERIALS*	2010	Article	10.1016/J.BIOMATERIALS.2010.01.113
2	116	Ophthalmic drug delivery systems--recent advances	LE BOURLAIS C	*PROG RETIN EYE RES*	1998	Review	10.1016/S1350-9462(97)00002-5
3	114	In vitro uptake and release studies of ocular pharmaceutical agents by silicon-containing and p-HEMA hydrogel contact lens materials	KARLGARD CCS	*INT J PHARM*	2003	Article	10.1016/S0378-5173(03)00124-8
4	111	Ocular drug delivery	GAUDANA R	*AAPS J*	2010	Review	10.1208/S12248-010-9183-3
5	110	Ophthalmic drug delivery through contact lenses	GULSEN D	*INVEST OPHTH VIS SCI*	2004	Article	10.1167/IOVS.03-0959
6	107	Ocular release of timolol from molecularly imprinted soft contact lenses	HIRATANI H	*BIOMATERIALS*	2005	Article	10.1016/J.BIOMATERIALS.2004.04.030
7	105	Modeling Ophthalmic Drug Delivery by Soaked Contact Lenses	LI CC	*IND ENG CHEM RES*	2006	Article	10.1021/IE0507934
8	99	The use of mucoadhesive polymers in ocular drug delivery	LUDWIG A	*ADV DRUG DELIVER REV*	2005	Review	10.1016/J.ADDR.2005.07.005
9	89	Imprinted soft contact lenses as norfloxacin delivery systems	ALVAREZ-LORENZO C	*J CONTROL RELEASE*	2006	Article	10.1016/J.JCONREL.2006.05.003
10	88	Dispersion of microemulsion drops in HEMA hydrogel: a potential ophthalmic drug delivery vehicle	GULSEN D	*INT J PHARMACEUT*	2005	Article	10.1016/J.IJPHARM.2004.11.033

## Data Availability

The original contributions presented in this study are included in the article. Further inquiries can be directed to the corresponding author.
